# The Role of MicroRNAs in Genome Response to Plant–Lepidoptera Interaction

**DOI:** 10.3390/plants8120529

**Published:** 2019-11-20

**Authors:** Katarína Ražná, Ľudovít Cagáň

**Affiliations:** 1 Department of Genetics and Plant Breeding, Slovak University of Agriculture, 94976 Nitra, Slovakia; 2 Department of Plant Protection; Slovak University of Agriculture, 94976 Nitra, Slovakia; ludovit.cagan@gmail.com

**Keywords:** RNA interference, microRNA, host–lepidoptera interaction, RNAi technologies

## Abstract

RNA interference is a known phenomenon of plant immune responses, involving the regulation of gene expression. The key components triggering the silencing of targeted sequences are double-stranded RNA molecules. The regulation of host–pathogen interactions is controlled by miRNA molecules, which regulate the expression of host resistance genes or the genes of the pathogen. The review focused on basic principles of RNA interference as a gene-silencing-based defense mechanism and the role of miRNA molecules in insect genomes. RNA interference as a tool for plant protection management is discussed. The review summarizes current miRNA-based biotechnology approaches for plant protection management.

## 1. Introduction: The Role of RNA Interference in Plant Immune Systems

Three types of RNA play a role in the process of transcription and translation of genetic information: messenger RNA, transfer RNA, and ribosomal RNA. Messenger RNAs are carriers of genetic information after transcription from a DNA molecule and are translated into proteins, whereas tRNAs and rRNAs are involved in the process of mRNA translation [1]. Small RNAs are not translated into proteins; they exhibit a wide spectrum of biological functions. Instead, these short nucleotide sequences regulate various biological processes in plants, animals, and humans [2]. There are two major categories of small RNAs: microRNAs (miRNAs) and small interfering RNAs (siRNAs) [3]. MiRNAs are 21–24 nt long and are generated from hairpin-like structures of RNA, and siRNAs are generated from dsRNAs [4]. Although they are chemically similar, they have different genomic origins and precursor structure. Besides these, small non-coding RNAs (ncRNAs) can be divided based on their size and function into repeat-associated small interfering RNAs (rasiRNAs), small nuclear RNAs (snRNAs), small nucleolar RNAs (snoRNAs), and piwi-interacting RNAs (piRNAs) [5]. Because the function of small ncRNAs is associated with the Argonaute family, proteins are in this respect divided into the Dicer-dependent group, including miRNAs and siRNAs, and the Dicer-independent group, which includes piRNAs [6]. Small RNAs are essential during organism development as well as for stress responses. They also control transposable elements and chromatin states. Small RNAs can be produced in response to pathogen attack. Under these circumstances, they act in defense mechanisms by directing the destruction of the invader [7].

RNA interference or post-transcriptional gene silencing (PTGS) is the mechanism by which protein-coding genes are regulated. In plants, PTGS acts as an antiviral system [8]. RNAi also plays a role as a natural defense mechanism against transposable elements that affect genome stability [9]. The process of RNA interference occurs as a response to double-stranded RNA that mediates resistance to both endogenous parasitic and exogenous pathogenic nucleic acids. It is based on post-transcriptional gene silencing induced by double-stranded RNA (dsRNA) [10,11] or hairpin structured RNA (hpRNA) [12]. There are several RNA-silencing pathways based on the precursor source (dsRNA or hpRNA) and the functional target of the small RNA [12,13]: microRNA pathway, RNA-directed DNA methylation pathway, trans-acting small interfering RNA (tasiRNA) pathway, and exogenic RNA silencing pathway. The gene expression of the pathogenic organism is controlled and restricted by the gene-silencing factor. In eukaryotic organisms, gene silencing fulfils the role of an adaptive defense system, and is transmitted systemically through organisms in response to pathogen attack [11,14,15].

The molecular mechanisms of RNA interference are bound to the presence of double-stranded RNAs molecules, which may originate exogenously from pathogen attack in a host organism, or endogenously in the form of microRNA molecules [12,16]. The first step involves Dicer-like (DCL1) proteins and RNase-III-like enzyme activity that processes dsRNA molecules into 21–23 nt short-interfering RNA (siRNA) duplexes. These duplexes are unwound and one of the two strands becomes part of the RNA-induced silencing complex (RICS), which cleaves the homologous single-stranded mRNAs [10,16].

MiRNA-mediated gene silencing in plants fulfills the function of positive or negative immune response regulator and/or activator of a positive or negative regulator of immune defense [17]. The miRNA pathway of RNA interference includes endogenous microRNA molecules of non-coding character which are derived from *MIR* genes transcribed by RNA polymerase II into primary miRNA transcripts (pri-miRNA), which form stem–loop or hairpin structures that are processed into precursor molecules (pre-miRNA) [18]. In the subsequent steps of the silencing mechanism, it is possible to observe similarity with the above-mentioned process based on the presence of exogenous dsRNA. Pre-miRNA is digested by DCL1 to generate 21 nt long duplexes comprised of mature miRNA (guide strand) and miRNA* (miRNA passenger strand) [12]. MiRNA duplexes are transported from the nucleus into the cytoplasm where the mature miRNA (typically 21 nt in size) is incorporated into an Argonaute (AGO) family protein (primarily AGO1) RNA-induced silencing complex (RICS) [12]. Argonaute proteins, as effectors of sRNA-mediated regulation, contain several functional domains (PAZ, MID, and PIWI), each with different roles in small RNA processing [19]. Given their sequence relatedness, the Argonaute subfamily proteins associate with miRNAs and also with siRNAs, whereas the piwi subfamily proteins bind piRNAs. Consequently, the miRNA strand guides the cleavage-targeted mRNA sequences or inhibition of translation of complementary mRNA targets [20].

## 2. MicroRNA Biogenesis and Function in Plants versus in Insects

MicroRNA molecules (miRNAs) have several attributes which define their functions in plants. These noncoding molecules originate from transcription of *MIR* genes, which may be conserved across several plant species or be species-specific [21,22,23]. Their regulatory function is indisputable. Because of their ability to move between plant cells, they can regulate various cellular and developmental processes and organogenesis [20,24]. A significant potential lies in their regulatory function (as already mentioned), which is even underlined by the genomic organization of *MIR* genes and can be categorized as intergenic, intronic, and exonic [25]. Intergenic miRNAs can be monocistronic, having their own promoters, or polycistronic, with several miRNAs transcribed as a cluster with a shared promoter. Intronic miRNAs are found in the introns of protein-coding and noncoding genes. They are transcribed from the same promoter as their host genes and are present as a single miRNA or as a cluster of several miRNAs. Exonic miRNAs are not so widespread in comparison to either of the types above, and often overlap an exon–intron sequences of a noncoding gene. They are also transcribed by their host gene promoter and their maturation often excludes host gene function. The origin of *MIR* genes can be explained by two models, one of which presents the genes’ origin as a result of inverted duplications of target genes, and the other which suggests that these genes are a result of numerous random inverted repeats in the genome [19]. Many plant miRNAs play critical roles in nutrient homeostasis and in plant immune system response to abiotic stress and pathogen invasion via interactions with target mRNAs [26,27].

Small RNA molecules generally result from fragmentation of longer RNA sequences. MiRNA precursors are double-stranded RNA molecules (dsRNAs) that are cleaved and modified by enzymes and proteins. The miRNA biosynthesis process cannot be considered universal for all classes of miRNAs [28]. The individual steps of biosynthesis can be accomplished in different ways and influenced by different mechanisms for individual miRNAs. These specific differences in miRNA synthesis suggest variability in the level of post-transcriptional regulation of miRNA expression [29]. MiRNA molecules are accumulated in a spatiotemporal manner, which points to strict regulation of the biogenesis process [19].

Despite the high stability of sRNAs in plants and animals, biogenesis of miRNAs in plants requires a specific structure, the Dicer body, which is essential for the formation of mature miRNA molecules [30].

MiRNAs are made up of primary transcription products (pri-miRNA), which can be up to 700 nt in length in plants, and may contain more than one miRNA. One or more hairpin structures (pre-miRNAs) arise from the primary transcript. This process is catalyzed by Drosha and Dicer endonuclease in the animal genome, or by a Dicer-like enzyme in the plant genome. The hairpin structures are exported from the nucleus to the cytoplasm, where these precursor molecules are cleaved to a miRNA duplex, RNA-induced silencing complex (RICS), which negatively regulates gene expression by inhibiting gene translation or degrading coding mRNAs by a perfect or near perfect complement to target mRNAs [21,23,27,31]. Based on the level of sequence complementarity between miRNAs and their targets, there are three variations of miRNA-mediated gene regulation: degradation of mRNA, repression of translation, and miRNA-mediated mRNA disintegration [32]. The near-perfect base pairing between miRNAs and their targets in plant genomes and some animal genomes leads to cleavage of target mRNAs; however, perfect pairing can lead to translation repression but not mRNA degradation in plant genomes.

Cleavage of the stem–loop precursor to short, approximately 21 nt long miRNA–miRNA* duplexes is a hallmark of plant miRNAs [27]. These duplexes are intermediates of miRNA biogenesis and are incorporated into the silencing complex. The size and structural variability of stem–loop structures is higher in plants than in animals [33,34].

Recent advances suggest that miRNA-guided translation inhibition is a major component of miRNA activity [30]. MicroRNA molecules recognize their target RNA sequences based on specific base pairing. The result of this pairing is influenced by the degree of complementarity between the two sequences. If the base pairing is perfect or nearly perfect, cleavage of the target mRNA occurs [35].

In insects, mature miRNAs can arise from monocistronic, bicistronic, or polycistronic miRNA transcripts [35], because several miRNA genes exist in form of gene clusters. Most of the miRNAs are generated from intergenic regions of the insect genome; however, some miRNA genes are located in intronic regions and are therefore transcribed together with genes of host organism [36]. RNA polymerase II mediates the transcription of miRNA loci into pri-miRNA transcripts. The primary transcripts may contain one or more stem–loop structures, which are processed into precursor miRNA (pre-miRNA) molecules by protein complexes or RNase-II-type enzymes Drosha and Pasha. Subsequently, pre-miRNA molecules are transported from the nucleus into the cytoplasm by exportin, where the stem–loop structure of pre-miRNA is cleaved by another RNase III enzyme, Dicer-1 (DCL 1), into short (22 nt) miRNA duplexes. Due to cleavage of the miRNA–miRNA* duplex, one strand is incorporated into the RNA-induced silencing complex (RICS), of which the main component is the Argonaute family of sRNA-guided RNA-binding proteins [35]. For the majority of insect miRNAs, the miRNA–AGO complex mediates regulation of target sequences; however, some miRNA require additional processing after AGO loading. Five different types of AGO protein have been recognized in *Drosophila genome* [36]. Most target mRNA plants contain only one complementary site for miRNA sequences, and most corresponding miRNAs perfectly complement these sites and cleave target mRNAs [23,35].

## 3. The Function of MicroRNA in Insect Genomes

Plant–insect interaction is very complex. On one hand, plants need to defend themselves against pathogens, but on other, they need to attract beneficial insects [32]. Several studies point to the involvement of miRNAs in these reciprocal interactions [32,37,38]. Together, more than 3000 insect miRNAs have been identified in insect species [31]. MiRNAs influence insect growth and development, and their ability to detoxify plant allelochemicals or insecticides. MiRNAs also have important roles in the regulation of mutual plant and insect physiological processes, such as, for example, insect oogenesis and embryogenesis, or host–pathogen interactions [39,40]. Plant small RNAs acquired orally through food intake are able to migrate through the plasma and directly influence gene expression in animals [7].

In Lepidopteran insects, a pest of maize, 58 putative miRNAs have been identified in *Spodoptera litura* [41]; 163 conserved and 13 novel miRNAs from *Manduca sexta* [42]; 127 conserved miRNAs in *Spodoptera exigua* [43]; 97 conserved miRNAs in *Helicoverpa armigera*; 91 conserved miRNAs in *Spodoptera litura*; and 127 conserved miRNAs in *Spodoptera exigua* [44]. According to evolutionary analysis, most of the identified miRNAs are insect-specific, and some are Lepidoptera-specific [45]. The potential role of miRNA in the evolution of *Bt* resistance is not yet clear [46]. In the midgut of Cry1Ab-susceptible and -resistant *Ostrinia nubilalis*, 277 miRNAs (248 conserved and 29 novel) have been discovered. This suggests that miRNAs play some role in these traits. Additionally, herbivores show transcriptional responses to specific plant structures [46]. In the case of *Bt* maize, further research about RNAi-based insecticides has been done focusing on coleopteran *Diabrotica virgifera virgifera* [47].

It has also been argued that polyphagous and monophagous insects probably have different approaches to plant defense. Generalists need to use a wide spectrum of strategies to detoxify plant defense systems; on the other hand, specialists need to be adapted to a specific host [35]. Generalists such as *H. armigera* or *M. sexta* larvae (both Noctuidae, Lepidoptera) consume the tissues of many different plants, and for the many different defensive host plant genes they need many different enzymes for detoxification or digestion [48]. This may be the reason for the high number of conserved miRNAs found in polyphagous lepidopterans attacking maize.

Plant defense is probably not restricted only to RNAs originating from the nucleus. It has been found that the weights of *H. armigera* developmental stages are influenced by chloroplast-derived dsRNAs [49]. Thus, insect–plant interactions based on miRNAs take on another dimension.

It has been found that plants react in different ways if they are mechanically injured compared to if they are injured by insects [50,51], and they react to the oral secretions of insects [52]. Thus, feeding of the *O. nubilalis* larvae induces higher levels of defensive compounds (benzoxazinoids, kauralexin family, diterpenoid phytoalexins) in maize stem tissues [53]. One survey of maize miRNA genes showed that mature miRNA genes were highly conserved during their evolution [54], with a total of 89 miRNA targets involved in different development or metabolism processes [55]. It has been suggested that small RNAs regulate gene expression and immune response [56], and could be utilized in plant protection [57]. Secretions of *M. sexta* alter the small RNA transcriptome [58], and the secretions of *S. exigua* cause rapid changes in gene expression 4–6 h after infestation of maize by larvae [59]. This means that both insects and plants react to each other during their interactions.

In some cases, it seems that insect attack may have partial positive effects on the nutritional content of maize stems. It was found that damage to maize stems caused by *O. nubilalis* influenced the defense of the plant, but the plant also excreted auxin indole-3-acetic acid (IAA) in its grass, which could be associated with increased levels of nutritional substances near pest tunnels in the stems [60].

## 4. RNA Interference as a Tool for Plant Protection Management

The increasing demand for safe strategies for crop improvement and plant protection management makes RNAi technology an attractive field of research. Because of insect resistance, the future of transgenic *Bt* crops is at risk. RNAi technologies therefore have the potential to provide an eco-friendly biotechnological approach [9].

Currently, there is increasing information about the resistance of *Bt* plants to insect pests, and it seems that RNAi will be the way to resolve the situation. It was identified that 20% of field-collected *H. armigera* males had *Bt* cotton resistance alleles [61]. Artificial microRNA targeting the ecdysone receptor (*EcR*) gene was used as possible alternative [62]. A strong resistance to Cry1Ab can also develop in *Ostrinia furnacalis*, a pest of maize in Asia, and differential expression of miRNAs especially to targeting potential *Bt* receptor genes has been found in its larvae [63]. Similarly, larval mortality of the pest can be increased by combined treatment with *Bt* toxin and dsRNAs [64].

*Diabrotica virgifera virgifera* belongs to the coleopteran species that feed on the maize plant. It has been found that ingestion of double-stranded dsRNAs may result in retarded larval development and mortality [65].

Recent observations have suggested that insects’ diets can influence their control of RNAi [66,67]. Studies have demonstrated that ingestion of dsRNAs supplied in an artificial diet triggers RNA interference in several coleopteran species [9,65].

Feeding of insect larvae with RNAi has been found to influence morphological aberrations accompanied with the arrest of molting in *H. armigera* [68], suppression of transcript degradation or translational repression in *H. armigera* [69], potential roles of miRNAs in *H. armigera* protease gene regulation [70], or abortive molting in *S. exigua* [43]. It has been suggested that gene suppression of *H. armigera* via RNA interference (RNAi) could be used for the management of this pest [71]. Diet experiments have been supported by experiments based on the adaptations of insect pests to their host plants. Two strains of *Spodoptera frugiperda* (a corn strain and a rice strain) differentially expressed miRNAs in the plant diet based on two host plants [72]. Two races of *O. nubilalis* are found in Europe (sibling species *O. nubilalis* and *O. scapularis*, with common ancestry). One race feeds mostly on maize, and the second feeds mainly on mugwort (*Artemisia vulgaris*) and hops (*Humulus lupulus*). *O. nubilalis* is specialized to maize, but *O. scapularis* remains polyphagous [73]. Expression of dsRNA targeted to suitable genes may provide alternatives for insect pest control [74] and pave the way for future pest control strategies. All these results highlight the influence of host plants on insects and suggest that miRNAs have some role in this process. There are more challenges to widespread use of RNAi [75], but probably one of the most important is their role in the potential development of resistance in insect populations.

## 5. The Potential of MiRNA-Based Biotechnology Approaches in Crop Response Improvement to Biotic Stress Factors

Research has shown that miRNA molecules play an important role in the genome responses of plants to stress factors. Many stress-regulated genes have been found to be regulated by miRNAs [76,77,78,79]. Conserved classes of miRNA molecules have been identified that exhibit the same responses to biotic stress conditions in different plant species. Studies show that by regulating the expression of a particular type of miRNA molecule, plant tolerance or increase in tolerance to a defined stress factor may be increased or decreased [80,81,82]. For examples, the upregulation of miR393, miR319, miR156, and others was observed after infection of *Arabidopsis* leaves with *P. syringae Pv*. *tomato* [83]. The increased activity of miR393 induced by pathogen-mediated suppression of auxin receptors leads to enhanced resistance to bacterial infection [84].

Several miRNAs have been identified that regulate viral resistance and, consequently, expression of virus-specific artificial miRNAs could provide novel approaches to crop resistance improvement [80].

MicroRNA research has multiple applications in the field of plant biotechnology (a) in the form of molecular markers based on miRNA sequences or (b) in the form of molecular breeding based on miRNA molecules. In both cases, the goal is to improve plant characteristics and properties [85]. Currently, miRNA molecules are being addressed as biotechnological tools to improve plant biomass, crop, and tolerance to biotic and abiotic environmental factors [86]. This knowledge will be important in improving plant tolerance to environmental stress conditions and understanding plant responses to given molecular conditions.

**Figure 1:**
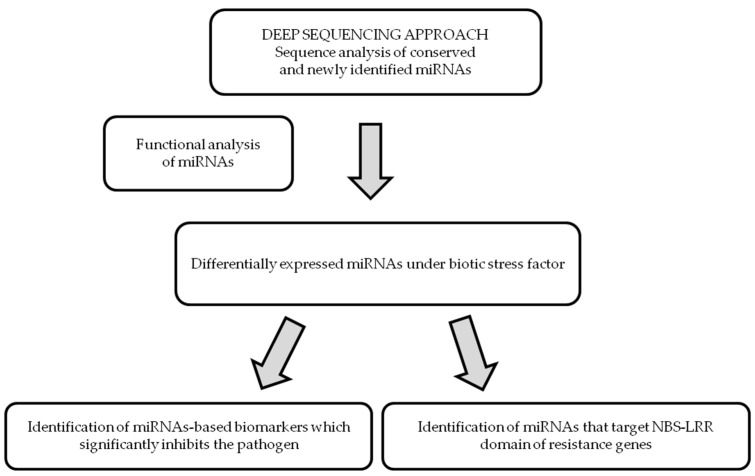
Methodological platform of miRNA-based strategies in crop improvement to biotic stress factors. Note: NBS-LRR - nucleotide-binding site (NBS)–leucine-rich repeat (LRR).

Improvement of plant tolerance to biotic stress factors is necessary in order to ensure quality and safe food resources. Progress in this area is also determined by the extent to which we have identified the molecular mechanisms of resistance. As miRNAs are the key players in plant responses to pathogen attack, knowledge of their functional role and regulation of their expression will be able to improve crop tolerance to biotic stress factors (Figure 1).

In addition to high-throughput sequencing procedures, technologies for identifying the function of miRNA molecules and their target sequences include modifying miRNA expression by applying miRNA inhibitors and generating mutant plants carrying a non-functional *MIR* gene. Another strategy to study the function of individual miRNA molecules is to apply “artificial” miRNAs (amiRNAs, artificial miRNAs) [87]. These molecules are designed to specifically target mRNA expression. The use of artificial miRNAs designed to suppress target genes represents a valuable approach for crop improvement [88]. It has been shown that regulating the expression of a single miRNA can enhance or decrease plant tolerance to abiotic stress factors (e.g., drought, salinity) [79]. An integral part of microRNA research, so-called “miRNomics” involves computer approaches to function prediction using bioinformatics tools (in silico procedures). 

## 6. Conclusions

The aim of the review was to highlight the key role of miRNA molecules during plant–lepidoptera interactions within plant immune system acceleration as a response to stress factors. RNA interference, which includes small RNA molecules, acts in the defense mechanisms by which the organism resists biotic stress factors. In the insect genome, miRNAs affect the growth and development of insects. MiRNAs are part of the insect regulatory mechanisms associated with plant detoxification. The host’s endogenous small RNA, as well as the RNA-silencing mechanism of the host organism, constitutes the basic level of control of the plant’s immune response. Recent studies show significant potential of RNAi biotechnologies. DsRNA fed as a diet component can be effective in downregulating targeted genes. Further knowledge of the mechanism and function of miRNA molecules in the regulation of plant defense mechanisms as well as in the genome of insects opens up new possibilities for environment friendly plant protection management.
